# Using ^18^F-FDG PET/CT-derived body composition features to predict lymphovascular invasion in non-small cell lung cancer

**DOI:** 10.1007/s00259-025-07435-4

**Published:** 2025-07-23

**Authors:** Zewen Jiang, David Haberl, Clemens Spielvogel, Szabolcs Szakall, Peter Molnar, Josef Yu, Victor Lungu, Janos Fillinger, Ferenc Renyi-Vamos, Clemens Aigner, Balazs Dome, Christian Lang, Lukas Kenner, Zsolt Megyesfalvi, Marcus Hacker

**Affiliations:** 1 Department of Biomedical Imaging and Image-Guided Therapy, Division of Nuclear Medicine, Medical University of Vienna, Währinger Gürtel 18-20, 1090 Vienna, Austria; 2 Christian Doppler Laboratory of Applied Metabolomics, Medical University of Vienna, Vienna, Austria; 3 Pozitron PET/CT Center, Budapest, Hungary; 4 National Koranyi Institute of Pulmonology, Budapest, Hungary; 5 Department of Thoracic Surgery, National Institute of Oncology-Semmelweis University, Budapest, Hungary; 6 National Institute of Oncology and National Tumor Biology Laboratory, Budapest, Hungary; 7 Department of Thoracic Surgery, Comprehensive Cancer Center, Medical University of Vienna, Vienna, Austria; 8 Department of Translational Medicine, Lund University, Lund, Sweden; 9 Division of Pulmonology, Department of Medicine II, Medical University of Vienna, Vienna, Austria; 10 Clinical Institute of Pathology, Medical University of Vienna, Vienna, Austria; 11 Unit of Laboratory Animal Pathology, University of Veterinary Medicine Vienna, Vienna, Austria; 12 Department of Molecular Biology, Umeå University, Umeå, Sweden; 13 Comprehensive Cancer Center, Medical University Vienna, Vienna, Austria

**Keywords:** NSCLC, Lymphovascular invasion, Body composition, ^18^F-FDG PET/CT

## Abstract

**Abstract:**

Lymphovascular invasion (LVI) in non-small cell lung cancer (NSCLC) is a critical prognostic marker linked to higher risks of metastasis and recurrence. This study aimed to develop a non-invasive predictive model using body composition features from ^18^F-FDG PET/CT imaging to assess LVI risk in early-stage NSCLC patients.

**Methods:**

We retrospectively analyzed 248 patients, including 153 from Vienna (training cohort) and 95 from Budapest (validation cohort). Preoperative ^18^F-FDG PET/CT scans were used to assess tumor metabolic parameters, including standardized uptake values (SUVmax, SUVmean), metabolic tumor volume (MTV), and total lesion glycolysis (TLG), as well as body composition features, including visceral, subcutaneous, and intermuscular adipose tissue, skeletal muscle at L1–L5. LASSO regression identified key body composition features, and a logistic regression-based nomogram was constructed and validated through ROC analysis, calibration, decision curve analysis, and survival analysis.

**Results:**

LVI was present in 66/153 (43.1%) of Vienna and 39/95 (41.1%) of Budapest patients. The nomogram, developed using the Vienna training cohort, incorporating MTV, N stage, and body composition achieved an AUC of 0.839 and 0.790 in the Budapest validation cohort. Statistical tests confirmed that the nomogram significantly outperformed models based on either clinical (*p* = 7.92e-06) or imaging variables alone (*p* = 0.0474). Furthermore, LVI predicted by the nomogram was associated with significantly poorer 3-year recurrence-free and 5-year survival.

**Conclusion:**

Integrating body composition with clinical and tumor metabolic features from PET/CT enables preoperative prediction of LVI in NSCLC, supporting improved risk stratification.

**Graphical abstract:**

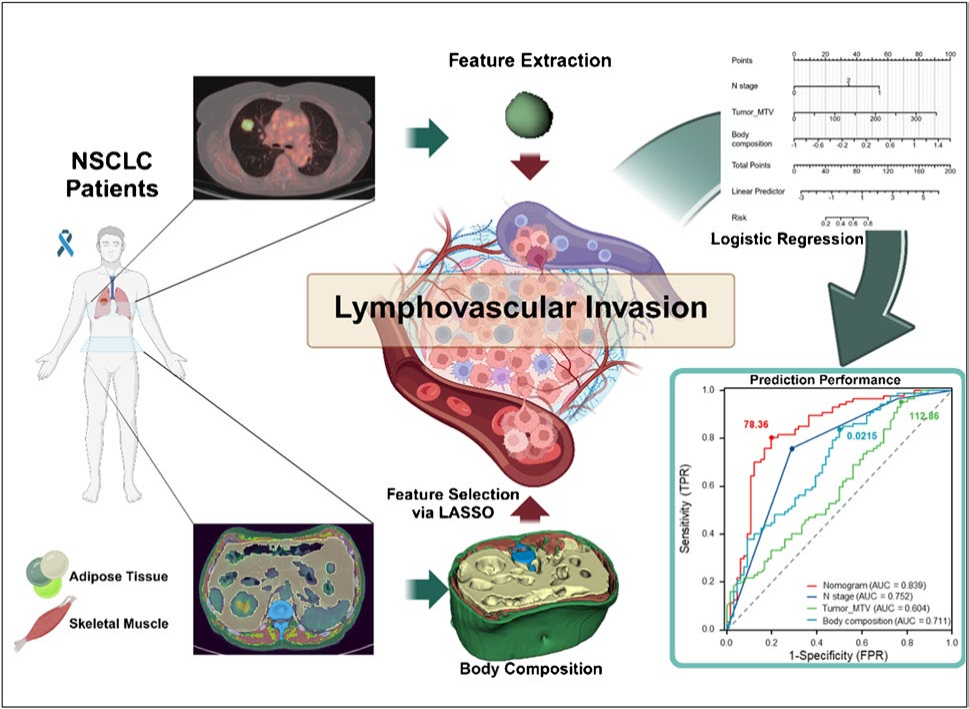

**Supplementary Information:**

The online version contains supplementary material available at 10.1007/s00259-025-07435-4.

## Introduction

Lung cancer is the second most commonly diagnosed cancer and the leading cause of cancer-related deaths, with non-small cell lung cancer (NSCLC) comprising 85% of cases [1, 2]. Despite various therapies, the prognosis for NSCLC remains poor with a 5-year survival rate around 25% [1, 3]. Surgical resection offers the best chance for a cure in early-stage NSCLC, yet relapse rates range from 30–55%, leading to significant mortality within five years [4]. Lymphovascular invasion (LVI), defined as the presence of tumor cell emboli within endothelial-lined lymphatic or vascular lumens [5], is a well-established predictor of poor outcomes due to its association with metastasis and recurrence [6–9] and may also support the consideration of neoadjuvant or adjuvant chemotherapy in selected high-risk cases, with the goal of improving recurrence-free (RFS) and overall survival (OS) [10, 11]. However, preoperative detection of LVI remains challenging, as confirmation currently relies on postoperative histopathology, with no validated non-invasive methods available.

Positron emission tomography-computed tomography (PET/CT) with ^18^F-fluoro-2-deoxy-d-glucose (FDG) plays a key role in lung cancer staging, relapse assessment, and treatment response evaluation. Studies indicate that FDG uptake parameters such as metabolic tumor volume (MTV) and maximum standardized uptake values (SUVmax) correlate with tumor aggressiveness and LVI in NSCLC [12–15]. Machine learning-based PET radiomics approaches have also demonstrated promising results in LVI prediction [12, 16], though clinical adoption remains limited and requires further validation. Recent evidence suggests that body composition, particularly the metabolic activity of adipose and muscle tissues assessed via PET/CT, may be an important prognostic factor in cancer [17]. These measurements reflect a patient’s nutritional status and serve as strong predictors of clinical outcomes [18, 19]. Body composition alterations, including skeletal muscle and fat loss, are common in cancer cachexia and negatively impact NSCLC prognosis [20]. Additionally, certain adipose and muscle tissue changes contribute to cancer progression by modulating inflammation and hormone levels [21, 22]. Emerging studies indicate that these metabolic alterations may also be associated with LVI in solid tumors, including primary liver cancers [23, 24].

Given this background, we hypothesized that the body composition of NSCLC patients may be related to LVI. This study aims to explore these associations and evaluate their potential for improving preoperative risk stratification in patients with NSCLC.

## Materials and methods

### Study population and follow-up

Retrospective data were collected from 248 NSCLC patients who underwent primary tumor resection at Vienna General Hospital (*n* = 153) and the National Korányi Institute of Pulmonology, Budapest (*n* = 95) between 2010 and 2020. The Vienna cohort (median follow-up: 47 months) served as the training cohort, while the Budapest cohort (median follow-up: 52 months) was used for validation. Institutional review board (IRB) approval was obtained, with informed consent waivers from the Medical University of Vienna (ID 1649/2016) and the Hungarian Medical Research Council (52,614–4/2013/EKU).

Patients were included if they had pathologically confirmed LVI status, complete clinical data, and preoperative PET/CT scans within two weeks before surgery. Follow-up visits were conducted at 3–6 months intervals for 2–3 years and annually thereafter. Recurrence and survival were assessed using chest CT imaging as the primary diagnostic tool. The study endpoints were RFS and OS. Detailed inclusion/exclusion criteria are provided in Supplementary Fig. 1.

### Clinical, pathological, and imaging data collection

Clinical and pathological data, including age, gender, smoking history, chronic obstructive pulmonary disease (COPD) status, surgical approach, and adjuvant therapy, were retrieved from medical records. PET/CT imaging was used to determine tumor SUVmax, MTV, total lesion glycolysis (TLG), and tumor location, following AJCC/UICC 8th edition staging [3].

### PET/CT imaging and tumor segmentation

**Fig. 1:**
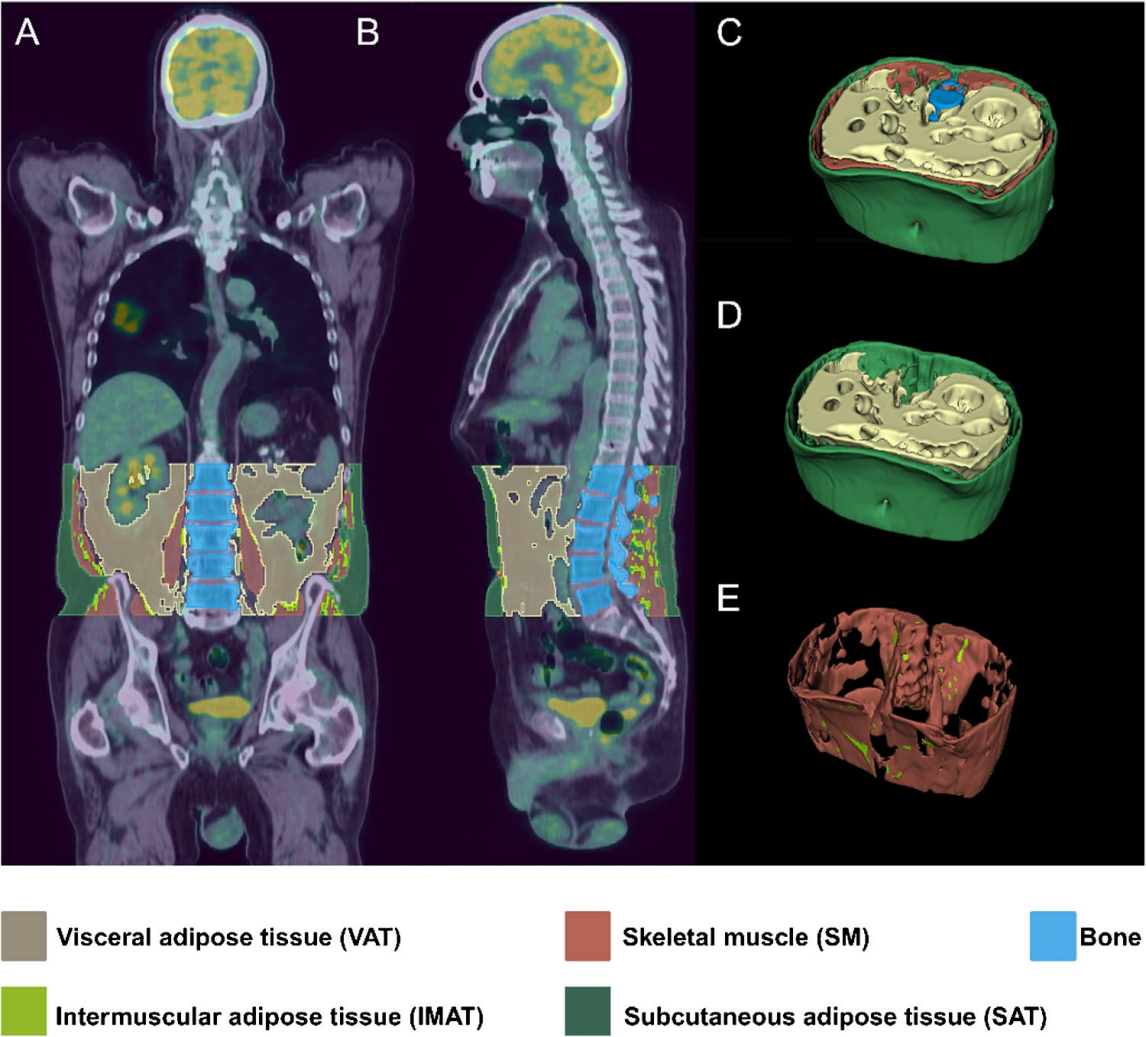
Automated body composition segmentation in NSCLC patients using ^18^F-FDG-PET/CT imaging. An example of automated segmentation of body compositions in an NSCLC patient is depicted using ^18^F-FDG-PET/CT images in the sagittal (**A**) and coronal (**B**) views. (**C**) A 3D rendering of all previously mentioned tissues, including visceral adipose tissue (VAT), subcutaneous adipose tissue (SAT), skeletal muscle (SM), and intermuscular adipose tissue (IMAT). (**D**) A 3D rendering of IMAT and SM. (**E**) A 3D rendering of SAT and VAT. The different colors represent the individual tissue classes: khaki for VAT, red for SM, blue for bone, green for IMAT, and bottle green for SAT

PET/CT imaging was performed for tumor metabolic assessment and body composition analysis. Imaging protocols followed standard guidelines to ensure consistency across cohorts. Tumor segmentation was conducted using AI-based methods and manual refinement for accuracy. Body composition features, including visceral adipose tissue (VAT), subcutaneous adipose tissue (SAT), intermuscular adipose tissue (IMAT), and skeletal muscle (SM) were extracted from CT scans [25], with corresponding SUV parameters assessed in adipose and muscle tissues. Figure 1 provides a visual representation of the segmentation process. Additional details regarding image acquisition and quantification can be found in the Supplementary Materials.

### Statistical analysis

Continuous variables were summarized as mean ± SD or median (IQR), while categorical variables were expressed as proportions. Wilcoxon rank-sum tests were used for continuous data comparisons, and chi-square tests for categorical data. LASSO regression was applied to select key body composition features for LVI risk stratification. Logistic regression models were constructed using clinical, tumor metabolic, and body composition features. A nomogram was developed from the multivariate model and validated using ROC curves, calibration plots, and decision curve analysis (DCA). Kaplan–Meier survival analysis was used to compare RFS and 5-year OS between high- and low-risk LVI groups using the log-rank test. Further statistical methods are detailed in Supplementary Materials.

## Results

### Baseline characteristics of patients

Baseline characteristics, including comparisons between the Vienna and Budapest cohorts, are presented in Table 1. LVI was confirmed in 66 patients (43.1%) from the Vienna cohort and 39 patients (41.1%) from the Budapest cohort. Both cohorts were similar in terms of age, BMI, COPD staus, and tumor location. However, significant differences were observed in gender distribution (*p* < 0.001), smoking status (*p* = 0.030), histological subtypes (*p* < 0.001), tumor differentiation (*p* < 0.001), and TNM stage (*p* < 0.001). Tumor metabolic parameters, including SUVmax, SUVmean, MTV, and TLG, were significantly higher in the Vienna cohort (all *p* < 0.001). Similarly, body composition features (VAT, SAT, SM, and IMAT) varied significantly between the cohorts, likely reflecting differences in patient demographics and metabolic profiles. Further analysis gender-specific differences were observed: male patients exhibited larger VAT and SM volumes compared to females in both cohorts (*p* < 0.001). Male patients also had higher SAT SUVmean (*p* < 0.001), while female patients exhibited higher VAT density and lower IMAT density (*p* < 0.05). These findings are further detailed in Table 1 and Supplementary Fig. 2.

**Table 1:** Baseline patient characteristics of the Vienna and Budapest cohorts

	Vienna 153	Budapest 95	*P*-value
Clinical and histopathology features			
Age, median (IQR)	64 (59, 71)	64 (59, 67.5)	0.175^1^
BMI, median (IQR)	26.172 (23.121, 29.412)	25.945 (23.842, 29.722)	1.000^1^
Gender, n (%)			< 0.001^2^
Female	65 (42.5%)	61 (64.2%)	
Male	88 (57.5%)	34 (35.8%)	
Smoking status, n (%)			0.030^2^
Never-smoker	50 (32,7%)	19 (20.0%)	
Current/ex-smoker	103 (67.3%)	76 (80.0%)	
COPD	84 (54.9%)	45 (47.4%)	0.248^2^
Histological subtypes, n (%)			< 0.001^2^
Adenocarcinoma	91 (59%)	95 (100%)	
Squamous cell carcinoma	62 (41%)	0 (0%)	
Tumor differentiation			< 0.001^3^
Well-differentiated	1 (0.7%)	3 (3.2%)	
Moderately differentiated	77 (50.3%)	72 (75.8%)	
Poorly differentiated	75 (49.0%)	20 (21.1%)	
Lymphovascular invasion, n (%)	66(43.1%)	39 (41.1%)	0.747^2^
Imaging features			
Tumor location, n (%)			0.315^2^
Right upper lobe	43 (28.1%)	32 (33.7%)	
Right middle lobe	16 (10.5%)	10 (10.5%)	
Right lower lobe	25 (16.3%)	15 (15.8%)	
Left upper lobe	45 (29.4%)	26 (27.4%)	
Left upper lobe	24 (15.7%)	12 (12.6%)	
T stage, n (%)			0.132^2^
T1	56 (36.6%)	33 (34.7%)	
T2	55 (35.9%)	46 (48.4%)	
T3	30 (19.6%)	13 (13.7%)	
T4	12 (7.8%)	3 (3.2%)	
N stage, n (%)			0.276^2^
N0	85 (55.6%)	62 (65.3%)	
N1	49 (32,0%)	22 (23.2%)	
N2	19 (12.4%)	11 (11.6%)	
TNM 8th stage			< 0.001^2^
I	46 (30.1%)	42 (44.2%)	
II	72 (47.1%)	37 (38.9%)	
III	35 (22.9%)	16 (16.8%)	
Tumor SUVmean, median (IQR)	5.0544 (3.172, 7.2809)	2.3971 (1.5078, 3.6473)	< 0.001^1^
Tumor SUVmax, median (IQR)	15.341 (8.4018, 22.584)	5.417 (2.6561, 7.3899)	< 0.001^1^
Tumor MTV, median (IQR)	25.429 (6.4195, 70.067)	7.616 (4.032, 19.552)	< 0.001^1^
Tumor TLG, median (IQR)	122.81 (23.213, 512.53)	19.483 (6.6883, 67.71)	< 0.001^1^
VAT cm3, median (IQR)	2532.8 (1462.6, 3610.7)	2338 (1545.2, 3495.9)	0.466^1^
SAT cm3, median (IQR)	3359.8 (2509, 4796.8)	3343.4 (2247.1, 4440.5)	0.486^1^
SM cm3, median (IQR)	2209.2 (1709.2, 2728.4)	1845.5 (1546.7, 2425.3)	0.004^1^
IMAT cm3, median (IQR)	137.59 (93.406, 218)	148.55 (116.98, 205.6)	0.366^1^
VAT HU, median (IQR)	−84.892 (−91.088, −70.297)	−89.758 (−96.011, −83.503)	< 0.001^1^
SAT HU, median (IQR)	−95.476 (−100.89, −88.596)	−97.146 (−102.51, −92.22)	0.163^1^
SM HU, median (IQR)	24.5 (16.484, 31.653)	23.017 (15.82, 28.923)	0.091^1^
IMAT HU, median (IQR)	−50.664 (−52.957, −48.964)	−51.393 (−53.472, −49.852)	0.025^1^
VAT SUVmean, median (IQR)	0.8186 (0.69413, 0.96911)	0.2417 (0.1838, 0.30183)	< 0.001^1^
SAT SUVmean, median (IQR)	0.3796 (0.31873, 0.45065)	0.47819 (0.38955, 0.54659)	< 0.001^1^
SM SUVmean, median (IQR)	0.68584 (0.58745, 0.80877)	0.50818 (0.45342, 0.64264)	< 0.001^1^
IMAT SUVmean, median (IQR)	0.62434 (0.52948, 0.72133)	0.51978 (0.42582, 0.6355)	< 0.001^1^
VAT SUVmax, median (IQR)	7.7321 (5.9643, 9.9299)	3.1474 (2.705, 3.9928)	< 0.001^1^
SAT SUVmax, median (IQR)	2.1485 (1.7793, 2.6231)	4.2118 (3.4337, 6.5617)	< 0.001^1^
SM SUVmax, median (IQR)	3.4083 (2.7521, 4.334)	3.7286 (3.2396, 4.9323)	0.013^1^
IMAT SUVmax, median (IQR)	2.2232 (1.8117, 2.7793)	3.1684 (2.6463, 4.2396)	< 0.001^1^

*Abbreviations*: *IQR* Interquartile range, BMI Body mass index, *COPD* Chronic obstructive pulmonary disease, *HU* Hounsfield Unit, *VAT* Visceral adipose tissue, *SAT* Subcutaneous adipose tissue, *IMAT* Intermuscular adipose tissue, *SM* Skeletal muscle, *SUV* Standardized Uptake Value, *MTV* Metabolic tumor volume, *TLG* Total lesion glycolysis. Median (IQR) was reported for continuous variables, and non-parametric tests were used to ensure robust comparisons between the Vienna and Budapest cohorts. The Wilcoxon rank-sum test^1^ was applied for continuous variables, while the Chi-squared test^2^, including Yates'correction^3^ where applicable, was used for categorical data

### Associations of body composition and tumor metabolism with histology subtypes, LVI, and pleural invasion in NSCLC

LASSO regression identified seven significant predictors of LVI from body composition features (Supplementary Fig. 3A-C). The LASSO-derived body composition risk score was computed as follows:

$$\begin{aligned} \text{Body composition score}=&-\mathrm{2,504106357}+{\mathrm{VAT}}_{\mathrm{cm}}^{3}\times \mathrm{0,000162631}\\&+{\mathrm{IMAT}}_{\mathrm{HU}}\times \mathrm{0,01935125}+{\mathrm{VAT}}_{\mathrm{SUVmean}}\\&\times \mathrm{0,648508908}+{\mathrm{SAT}}_{\mathrm{SUVmean}}\times \mathrm{0,488623834}\\&+{\mathrm{VAT}}_{\mathrm{SUVmax}}\times \text{0,203742721 }+{\mathrm{SAT}}_{\mathrm{SUVmax}}\\&\times\text{0,122565444 }+{\mathrm{IMAT}}_{\mathrm{SUVmax}}\times \mathrm{0,030738805} \end{aligned}$$

Further analysis revealed no significant correlations between body composition features (VAT, SAT, SM, IMAT, and the LASSO-derived body composition score) and tumor metabolic markers (SUVmean, SUVmax, MTV, and TLG) (Supplementary Fig. 4 A, B) However, body composition features showed associations with histological subtypes and pleural invasion. In the Vienna cohort, squamous cell carcinoma (SCC) exhibited significantly higher SUVmean and SUVmax in VAT, SAT, and IMAT compared to adenocarcinoma (ADC) (*p* < 0.05). Additionally, pleural invasion-positive cases demonstrated higher VAT SUVmax (*p* = 0.0047), with similar trends observed in the Budapest cohort (Supplementary Fig. 5).

### Logistic regression and nomogram development for LVI prediction

**Fig. 2:**
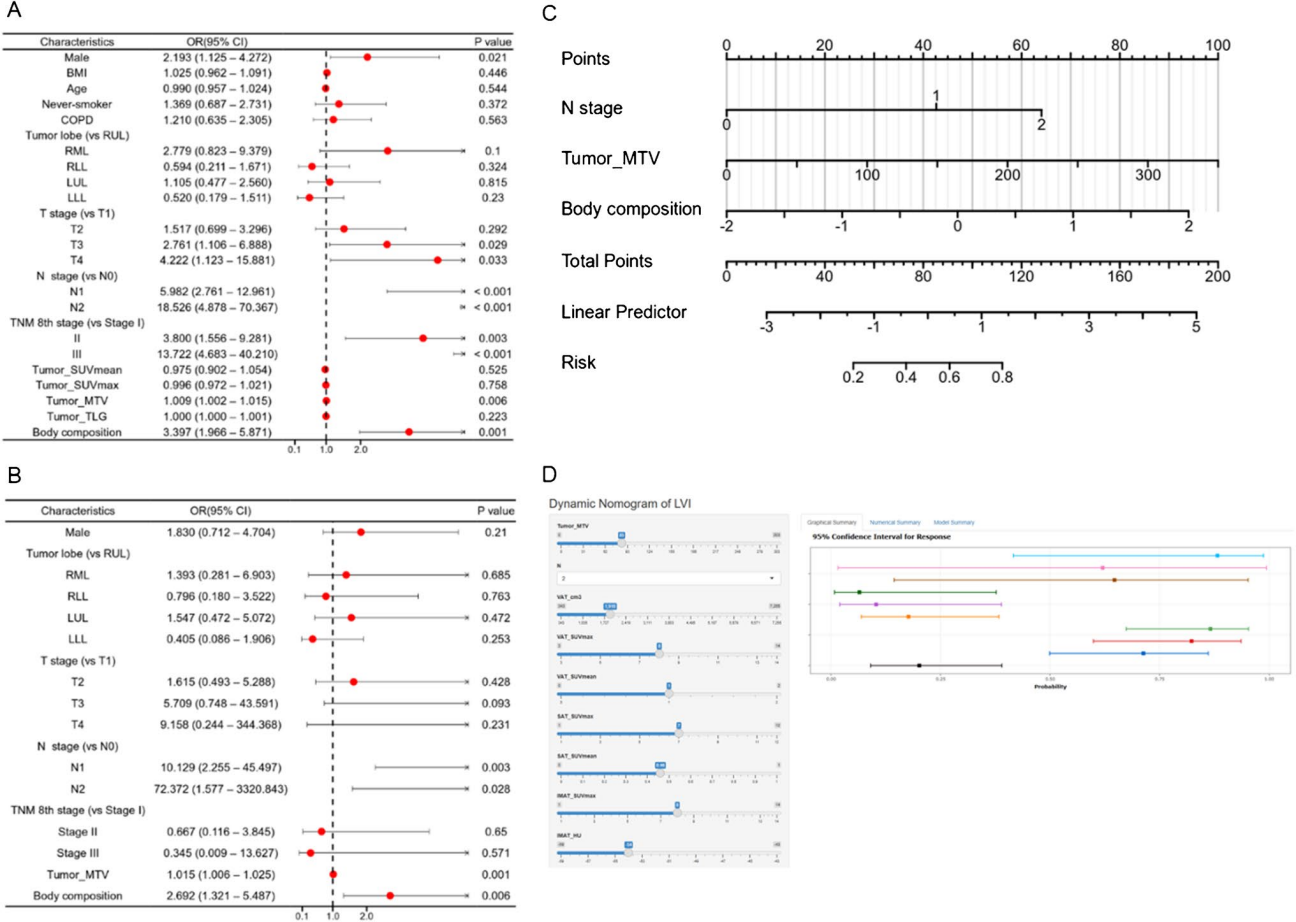
Forest plot and nomogram for LVI prediction. Forest plot and nomogram for Vienna dataset. The forest plot illustrates the development of the logistic regression model, presenting odds ratios (OR) and 95% confidence intervals (CI) from the binary logistic regression analysis. It includes the *p*-values for significance tests conducted in the univariate analysis (**A**) and the subsequent multivariate analysis (**B**). The nomogram was then constructed based on independent predictive factors, providing a visual representation of the risk prediction for LVI (**C**). A free browser-based online calculator based on nomogram predicting LVI (**D**). The left side of the figure shows the relevant risk factor options. Fill in the patient’s individual status according to the options, such as the N stage, the tumor MTV, and body composition score. The probability of postoperative LVI for the individual patient is generated after filling in the patient’s individual risk factor status, and the probability and 95% confidence intervals for response are shown on the right

Univariate and multivariate logistic regression analyses were performed to assess the predictive value of clinical and imaging features for LVI (Fig. 2A and 2B; details in Supplementary Table 1). N stage was identified as a significant independent predictor, with N1 (OR: 10.129, 95% CI: 2.255–45.497, *p* = 0.003) and N2 (OR: 72.372, 95% CI: 1.577–3320.843, *p* = 0.028) demonstrating a strong association with LVI. Tumor MTV (OR: 1.015, 95% CI: 1.006–1.025, *p* = 0.001) and body composition (OR: 2.692, 95% CI: 1.321–5.487, *p* = 0.006) were also identified as significant predictors. These three variables were incorporated into a nomogram for LVI prediction (Fig. 2C), which is available online for dynamic interaction at https://bodycomposition.shinyapps.io/dynnomapp/ (Fig. 2D).

### Evaluation of the predictive performance of the nomogram for LVI

**Fig. 3:**
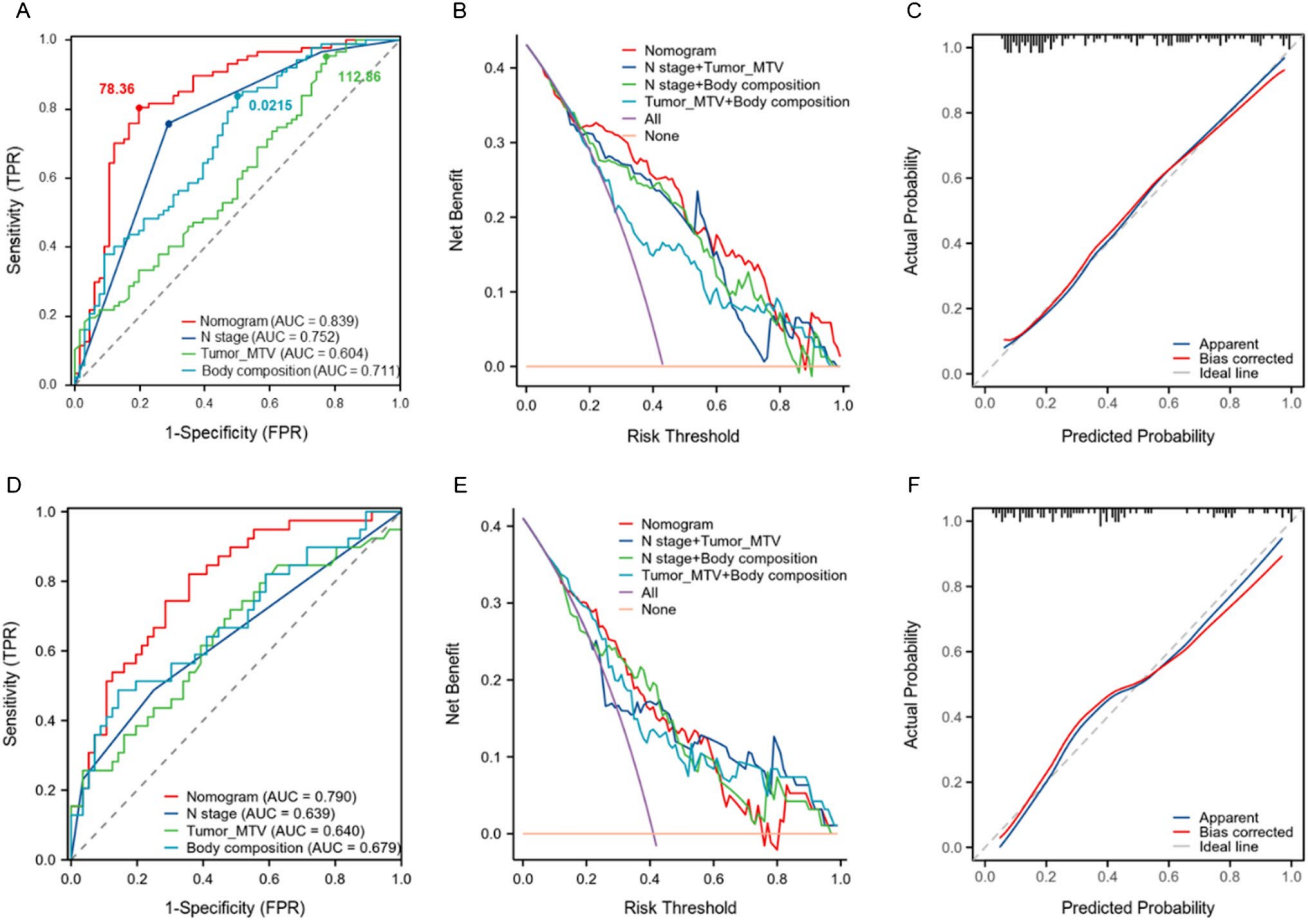
AUC comparisons, decision curve analysis, and calibration curves for the nomogram in the Vienna and Budapest cohorts. The comparisons of AUC between the nomogram and individual predictors in the Vienna training cohort (**A**) and Budapest validation cohort (**D**). Decision curve analysis (DCA) for the Vienna training cohort (**B**) and Budapest validation cohort (**E**). Calibration curves for the Vienna training cohort (**C**) and Budapest validation cohort (**F**), illustrating the agreement between observed and predicted probabilities

The nomogram demonstrated superior performance compared to individual predictors. In the Vienna cohort, the AUC values for the nomogram, tumor MTV + N stage, N stage, body composition, and tumor MTV were 0.839, 0.793, 0.752, 0.711, and 0.604, respectively. In the Budapest validation cohort, the AUC values were 0.790, 0.721, 0.640, 0.679, and 0.639, respectively (Fig. 3A, 3D; Supplementary Table 2). DCA confirmed the clinical utility of the nomogram by showing the highest net benefit across threshold probabilities (Fig. 3B, 3E). Calibration plots indicated strong agreement between observed and predicted LVI probabilities (Fig. 3C, 3 F).

The model's diagnostic performance was evaluated in both cohorts. In the Vienna cohort, the nomogram achieved 80.5% sensitivity, 80.3% specificity, and 80.4% overall accuracy. In the Budapest validation cohort, sensitivity, specificity, and accuracy were 23.1%, 95.0%, and 65.3%, respectively. DeLong's method confirmed the nomogram’s superior performance over tumor MTV (*p* = 0.0474) and N stage (*p* = 7.92e-06) (Supplementary Table 3). While the difference between the nomogram and body composition was not statistically significant (*p* = 0.0789), the nomogram still demonstrated higher predictive accuracy.

### Risk stratification of LVI based on the nomogram and survival analysis

**Fig. 4:**
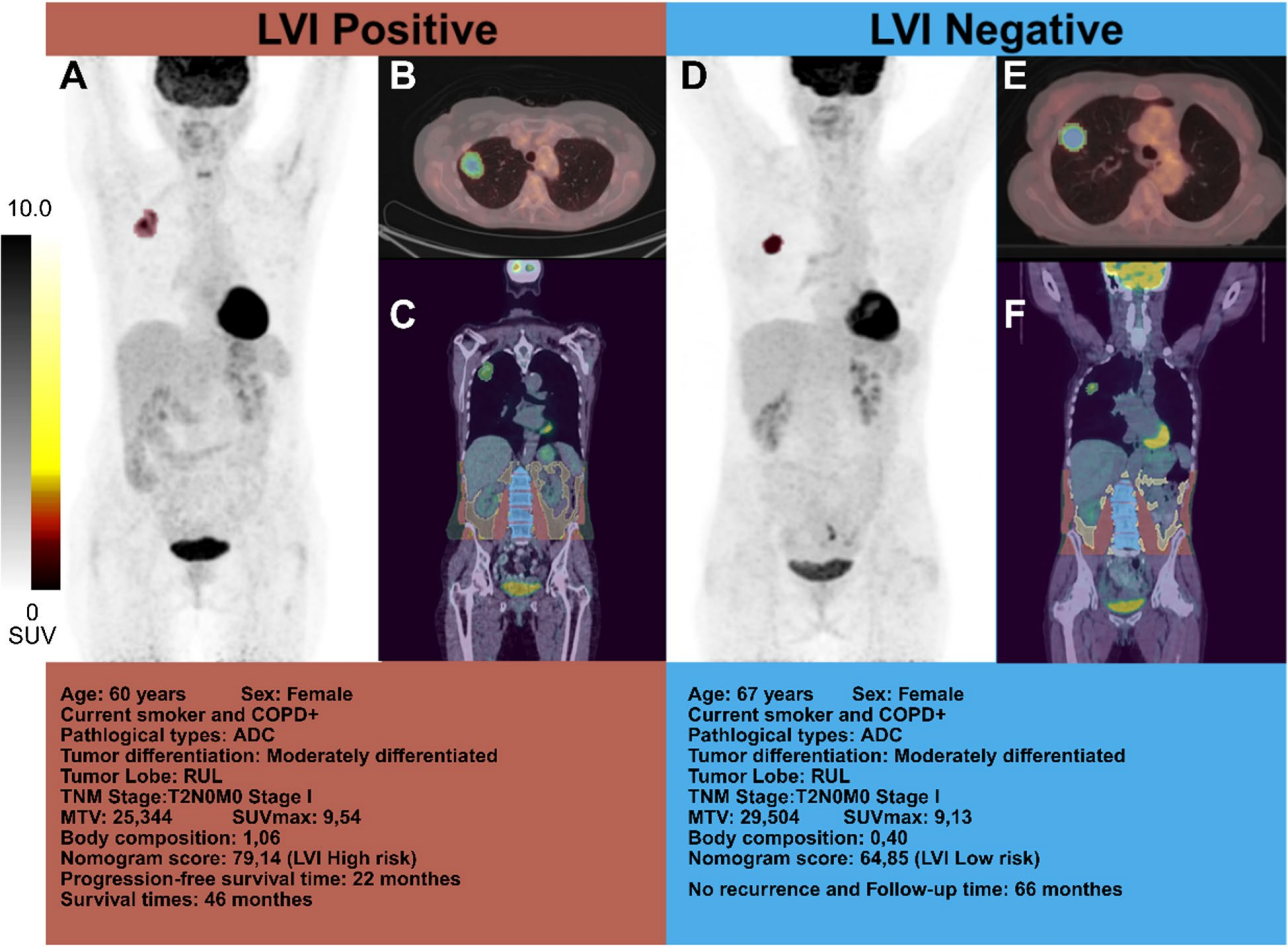
Two representative NSCLC patients with different LVI status. Representative imaging findings for LVI-positive (**A**–**C**) and LVI-negative (**D**–**F**) NSCLC patients. Panels show maximum intensity projection (MIP) images (A, D), fused ^18^F-FDG PET/CT coronal views of the lesion (B, E), and axial fused 18F-FDG PET/CT images highlighting body composition (C, F). The two patients had similar clinicopathological characteristics, including TNM stage and tumor differentiation. However, the LVI-positive patient experienced shorter progression-free survival (22 months) and overall survival (46 months), while the LVI-negative patient remained recurrence-free at 66 months of follow-up. These cases illustrate how differences in LVI status correspond to both imaging phenotypes and clinical outcomes

**Fig. 5:**
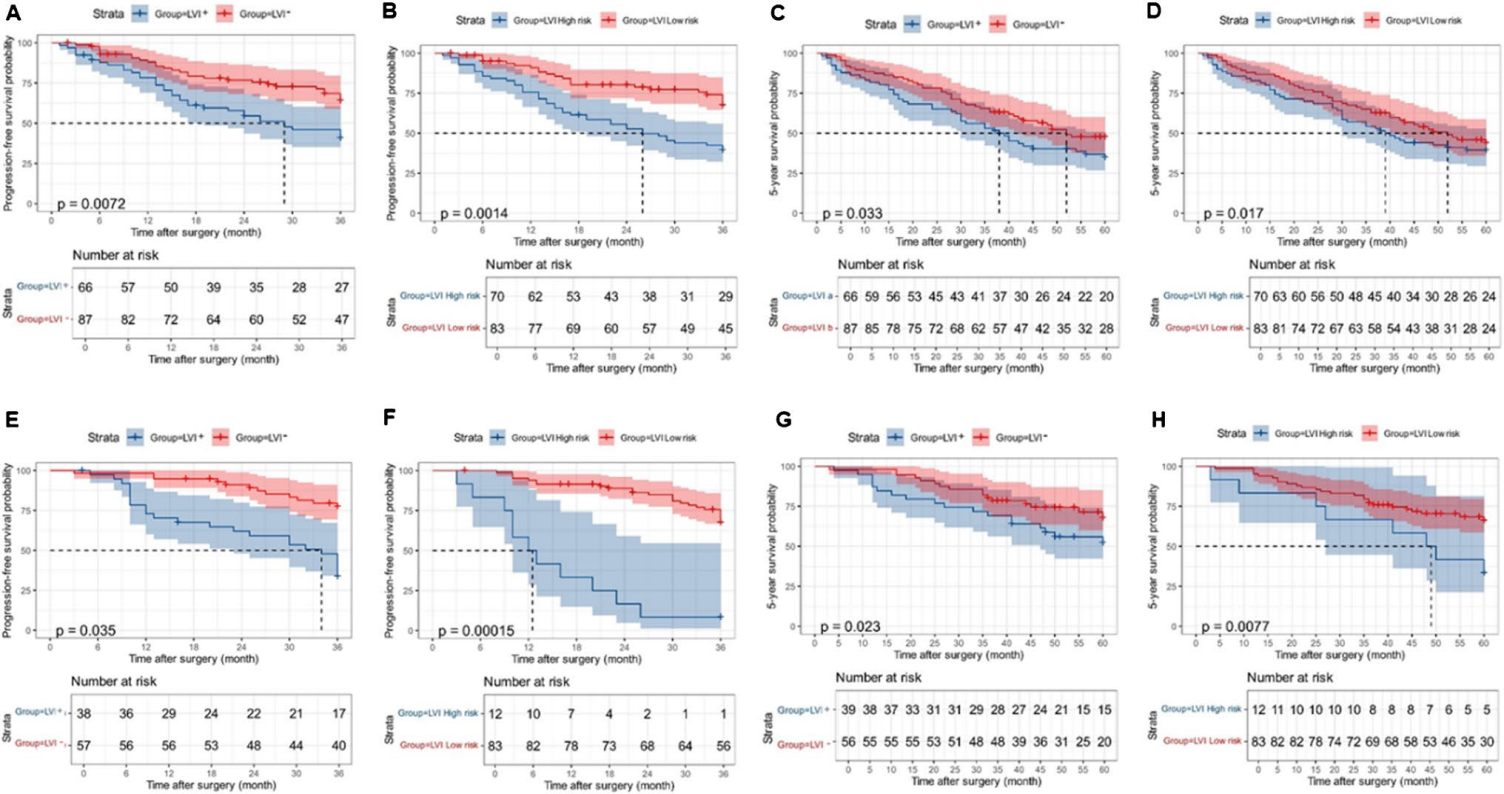
Prognostic value of pathological LVI status and LVI status predicted by the Nomogram. The Kaplan–Meier curves illustrate 3-year recurrence-free survival (RFS) and 5-year survival in the Vienna cohort (**A**–**D**) and the Budapest cohort (**E**–**H**), stratified by pathological LVI status and LVI status predicted by the nomogram

Using an optimal nomogram score cut-off of 78.36 (Fig. 3A), patients were categorized into high-risk (> 78.36) and low-risk (≤ 78.36) groups. To illustrate this risk stratification in individual cases, Fig. 4 presents two representative NSCLC patients with different LVI statuses, demonstrating variations in imaging characteristics and clinical outcomes. Kaplan–Meier survival curves showed that high-risk patients had significantly worse 3-year RFS and 5-year OS compared to low-risk patients in both Vienna (Fig. 5A-D) and Budapest (Fig. 5E-F) cohorts (*p* < 0.05). The prognostic value of N stage, tumor MTV, and body composition was confirmed in both cohorts (Supplementary Fig. 6). Subgroup analyses demonstrated that the nomogram effectively distinguished high- and low-risk LVI groups across different T-stage subgroups, confirming its robust predictive ability for both 3-year RFS and 5-year OS (*p* < 0.05) (Supplementary Fig. 7).

## Discussion

Body composition has emerged as a valuable predictor of clinical outcomes and prognosis in cancer patients, with imaging-based assessments providing detailed insights into fat and muscle tissue characteristics [17, 18]. LVI is a recognized predictor of early recurrence and poor prognosis [7, 9, 13]. However, most previous studies using AI have focused on extracting features from the tumor region, and combined clinical features to predict LVI in NSCLC [13, 26, 27]. To our knowledge, this study is the first to introduce a semi-automated 3D quantitative analysis of body composition as an independent predictor of LVI. The resulting non-invasive preoperative nomogram demonstrated high accuracy and strong predictive performance.

Multivariate analysis confirmed that MTV and N stage are independent LVI predictors, aligning with previous models [28–30]. ^18^F-FDG uptake is closely associated with several key tumor processes, including glucose metabolism (mediated by GLUT1 and hexokinase I), hypoxia response (via HIF-1α), angiogenesis (driven by VEGF and CD34), and the PI3K/Akt/mTOR signaling pathway, which promotes tumor invasiveness [31]. Studies show that LVI-positive patients also face poor outcomes, and accurate preoperative prediction of LVI is critical for guiding treatment decisions [32, 33]. Our analyses demonstrated that both histopathological LVI and nomogram-predicted LVI were significantly associated with 3-year PFS and 5-year survival, further confirming the clinical relevance of LVI as a prognostic factor. The nomogram provides an opportunity for risk stratification and informed clinical decision-making in NSCLC patients.

A key strength of this study is the integration of body composition features into the preoperative prediction of LVI, highlighting their association with clinical outcomes. However, the two cohorts differed significantly in several baseline characteristics, including gender, histological subtypes, TNM stage, and tumor metabolic parameters, limiting direct comparability. Despite these differences, the model maintained consistent predictive performance. Subgroup analyses further supported the nomogram’s robustness, demonstrating effective stratification of LVI risk across different T-stage groups.

Our LASSO regression model identified key fat-related (VAT cm^3^, VAT SUVmean, SAT SUVmean, VAT SUVmax, SAT SUVmax) and muscle-related (IMAT HU, IMAT SUVmax) features as significant LVI predictors. High VAT content promotes inflammation and tumor progression via cytokine secretion (leptin, resistin, IL-6, and TNF- α), angiogenesis, and insulin resistance [34–38]. VAT metabolic activity (SUVmean, SUVmax) correlates with glucose uptake and fatty acid release, fueling pro-tumor processes. Though VAT is more linked to metabolic disorders [39], SAT also contributes to inflammation and dysfunction, possibly supporting LVI development [40–42]. These factors may contribute to LVI development. Additionally, our findings indicate that higher SUVmean values in SAT and VAT are significantly associated with pleural invasion, further reinforcing the link between body composition and adverse outcomes in NSCLC patients. Muscle-related features, including IMAT HU and IMAT SUVmax, were also identified as significant predictors of LVI, highlighting their importance in cancer progression [43]. IMAT, representing fat infiltration within and between muscle cells, reflecting fat infiltration, poor muscle quality, and increased secretion of pro-inflammatory factors, which promote tumor proliferation [43–46]. These findings highlight the crucial role of fat and muscle-related features in predicting LVI.

Despite promising results, some limitations exist. A key challenge is the presence of baseline differences between the Vienna and Budapest cohorts, particularly regarding gender distribution, histological subtypes, tumor stage, and metabolic features. These differences reflect real-world clinical variability and may have influenced model sensitivity in the external validation. Gender distribution differed significantly between cohorts, with more female patients in Budapest. Male patients (more common in Vienna) had higher VAT and SM volumes as well as SAT SUVmean, contributing to higher body composition scores. These gender-related differences may influence model performance. Lacking of histological balance between the two cohorts presents another limitation. Another limitation is the imbalance in histological subtypes between cohorts. The Budapest cohort consisted exclusively of adenocarcinoma (ADC) patients, while the Vienna cohort included both squamous cell carcinoma (SCC) and ADC. As SCC is more glucose-dependent and metabolically distinct from ADC [47], this histological imbalance may have contributed to the higher MTV values observed in the Vienna cohort. Consequently, the median metabolic tumor volume (MTV) was notably higher in the Vienna cohort (25.4 cm^3^) than in the Budapest cohort (7.6 cm^3^), reflecting a metabolic difference between the two populations. However, histological subtype is typically unknown at the time of preoperative imaging, and the model was intentionally designed to function independently of such information.

Additionally, while the model demonstrated strong performance across both cohorts, the observed differences in cohort characteristics raise the possibility of overfitting. Variability in patient populations, particularly in tumor metabolic parameters and body composition, can impact model sensitivity. However, despite these differences, the validation model retained high specificity (95.0%) and a stable AUC (0.790), supporting its robustness across different populations.

Future research should aim to validate this nomogram in larger, multi-center NSCLC populations and explore additional imaging biomarkers to further enhance predictive performance. Investigating the biological mechanisms linking body composition to tumor progression could provide new therapeutic perspectives in NSCLC management.

## Conclusion

In conclusion, our study constructed a nomogram integrating body composition features-specifically VAT, SAT, SM, and IMAT-with clinical variables to predict LVI in NSCLC patients non-invasively. Patients with higher LVI risk scores may benefit from adjuvant therapies to reduce recurrence and improve survival outcomes. The model's strong predictive accuracy indicates its potential for guiding individualized treatment strategies and improving prognosis in NSCLC patients.

## Supplementary Information

Below is the link to the electronic supplementary material.

Supplementary file1 (DOCX 2992 kb)

## Data Availability

The datasets generated or analyzed during the current study are available from the corresponding author upon reasonable request.
