# Supplementary material for: Using 18F-FDG PET/CT-derived body composition features to predict lymphovascular invasion in non-small cell lung cancer
Source: Eur J Nucl Med Mol Imaging. 2025 Jul 23;53(2):774–85. doi: 10.1007/s00259-025-07435-4 (PMC12830411; doi:10.1007/s00259-025-07435-4)
Supplement: Supplementary file 1 — Supplementary file1 (DOCX 2992 kb) [file 259_2025_7435_MOESM1_ESM.docx]

**Supplement Data**

**Image Acquisition and Quantification**

In the Vienna cohort, ^18^F-FDG-PET/CT imaging was conducted using the Siemens Vision 600 scanner, while in the Budapest cohort, the Siemens Biograph TruePoint 6 HD PET/CT scanner was employed. Patients followed standard clinical preparation, including fasting for ≥6 hours to maintain blood glucose levels <150 mg/dL. For CT acquisition, non-contrast low-dose CT scans were performed for attenuation correction and anatomic correlation of the PET findings. PET images were reconstructed using standard clinical protocols specific to each scanner, with a voxel size of 4.0 mm×4.0 mm×4.0 mm, ensuring compliance with EARL guidelines (1). Image intensities were then converted to SUV normalized to body weight. Additionally, body composition features, including visceral adipose tissue (VAT), subcutaneous adipose tissue (SAT), intermuscular adipose tissue (IMAT), and skeletal muscle (SM), were extracted from CT scans at the L1–L5 vertebral level, with corresponding SUV parameters assessed in adipose and muscle tissues.

Tumor segmentation was performed using the open-source software 3D Slicer (version 5.4.0) (2). The primary tumor from two cohorts was initially segmented using nnU-Net (3), an artificial intelligence (AI)-based lesion segmentation model developed for automatic segmentation in medical imaging, followed by manual refinement to ensure accuracy. The region of interest (ROI) was manually refined by drawing polygonal boundaries to enclose the entire tumor, ensuring adequate margins on all slices where the tumor was visible while excluding physiological ^18^F-FDG uptake. SUVmax was the voxel with the highest FDG uptake within the ROI. SUVmean was calculated as the average of all voxels within the ROI. MTV was calculated from the entire tumor load, while TLG was calculated as the product of SUVmean and MTV (1).

For the body composition analysis, TotalSegmentator (v2.5.0-weights) (4) was used to automatically segment various organ classes, including adipose and muscle tissue, from L1 to L5 vertebral levels. Both tumor and body composition segmentations in two cohorts were manually refined by a single observer with over five years of clinical diagnostic imaging experience, under the supervision of a nuclear physician with more than ten years of experience to ensure accuracy and consistency. Volume (cm³) and density (HU) were recorded for each tissue type, and SUVmean and SUVmax were extracted from the corresponding segmentations.

**Detailed Statistical Methods**

Categorical variables were compared between LVI-positive and LVI-negative groups using chi-square tests, while continuous variables were assessed using t-tests or Mann-Whitney U tests depending on data distribution. The Shapiro-Wilk test was applied to assess normality. LASSO regression was used to reduce data dimensionality and select significant body composition features, with tenfold cross-validation optimizing the penalty parameter. Features with non-zero coefficients were selected to compute a LASSO-derived risk score for LVI prediction.

LASSO regression was used to reduce data dimensionality and select key body composition features, with tenfold cross-validation optimizing the penalty parameter. Features with non-zero coefficients were used to compute a LASSO-derived risk score for LVI prediction. Univariate and multivariate logistic regression analyses were performed to identify potential predictors of LVI, including clinical, tumor metabolic (SUVmax, SUVmean, MTV, TLG), and body composition features. Variables with p-values < 0.1 in univariate analysis were included in the multivariate model, and odds ratios (ORs), 95% confidence intervals (CIs), and p-values were calculated. A predictive nomogram was developed using significant variables from the multivariate model, with the Youden index determining the optimal cut-off point. This nomogram was externally validated using the Budapest cohort, applying the same coefficients from the Vienna cohort for consistency. Predictive performance was assessed using ROC curve analysis (AUCs calculated) and calibration plots, which evaluated the agreement between predicted probabilities and actual outcomes. Decision curve analysis (DCA) was also performed to assess clinical utility.

Kaplan-Meier survival analysis was used to generate survival curves for 3-year RFS and 5-year survival based on LVI risk stratification. Survival differences between high- and low-risk groups were compared using the log-rank test.

All analyses were conducted using R software (version 4.0.2) with the following packages: glmnet (LASSO regression), rms (nomogram development), pROC (ROC analysis), survival (Kaplan-Meier analysis), and ggplot2 (visualization).

| **Supplement Table 1. Univariate and multivariate analysis of variables associated with LVI in NSCLC.** | | | | |
| --- | --- | --- | --- | --- |
| Characteristics | OR (95% CI) Univariate analysis | P value | OR (95% CI) Multivariate analysis | P value |
| BMI | 1.025 (0.962 – 1.091) | 0.446 |  |  |
| Gender |  |  |  |  |
| Female | Reference |  | Reference |  |
| Male | 2.193 (1.125 – 4.272) | 0.021 | 1.830 (0.712 – 4.704) | 0.210 |
| Age | 0.990 (0.957 – 1.024) | 0.544 |  |  |
| Smoke |  |  |  |  |
| Current/ex-smoker | Reference |  |  |  |
| Never-smoker | 1.369 (0.687 – 2.731) | 0.372 |  |  |
| COPD |  |  |  |  |
| Negative | Reference |  |  |  |
| Positive | 1.210 (0.635 – 2.305) | 0.563 |  |  |
| Tumor location |  |  |  |  |
| Right upper lobe | Reference |  | Reference |  |
| Right middle lobe | 2.779 (0.823 – 9.379) | 0.100 | 1.393 (0.281 – 6.903) | 0.685 |
| Right lower lobe | 0.594 (0.211 – 1.671) | 0.324 | 0.796 (0.180 – 3.522) | 0.763 |
| Left upper lobe | 1.105 (0.477 – 2.560) | 0.815 | 1.547 (0.472 – 5.072) | 0.472 |
| Left lower lobe | 0.520 (0.179 – 1.511) | 0.230 | 0.405 (0.086 – 1.906) | 0.253 |
| Tumor SUVmean | 0.975 (0.902 – 1.054) | 0.525 |  |  |
| Tumor SUVmax | 0.996 (0.972 – 1.021) | 0.758 |  |  |
| Tumor MTV | 1.009 (1.002 – 1.015) | 0.006 | 1.015 (1.006 – 1.025) | 0.001 |
| Tumor TLG | 1.000 (1.000 – 1.001) | 0.223 |  |  |
| T Stage |  |  |  |  |
| T1 | Reference |  | Reference |  |
| T2 | 1.517 (0.699 – 3.296) | 0.292 | 1.615 (0.493 – 5.288) | 0.428 |
| T4 | 2.761 (1.106 – 6.888) | 0.029 | 5.709 (0.748 – 43.591) | 0.093 |
| T3 | 4.222 (1.123 – 15.881) | 0.033 | 9.158 (0.244 – 344.368) | 0.231 |
| N Stage |  |  |  |  |
| N0 | Reference |  | Reference |  |
| N1 | 5.982 (2.761 – 12.961) | < 0.001 | 10.129 (2.255 – 45.497) | 0.003 |
| N2 | 18.526 (4.878 – 70.367) | < 0.001 | 72.372 (1.577 – 3320.843) | 0.028 |
| TNM 8th stage |  |  |  |  |
| I | Reference |  | Reference |  |
| II | 3.800 (1.556 – 9.281) | 0.003 | 0.667 (0.116 – 3.845) | 0.650 |
| III | 13.722 (4.683 – 40.210) | < 0.001 | 0.345 (0.009 – 13.627) | 0.571 |
| Body composition | 3.397 (1.966 – 5.871) | < 0.001 | 2.692 (1.321 – 5.487) | 0.006 |

| **Supplement Table 2. Diagnostic performance of basic models in training and validation cohort.** | | |
| --- | --- | --- |
|  | **Vienna cohort** | **Budapest cohort** |
| Model | AUC (95CI%) | AUC (95CI%) |
| Nomogram | 0.839 (0.771 – 0.906) | 0.790 (0.699 – 0.881) |
| Tumor MTV+ N stage | 0.793 (0.717 – 0.869) | 0.721 (0.612 – 0.831) |
| Tumor MTV | 0.604 (0.513 – 0.694) | 0.639 (0.539 – 0.739) |
| N stage | 0.752 (0.681 – 0.824) | 0.640 (0.524 – 0.756) |
| Body composition | 0.711(0.628 – 0.793) | 0.679 (0.567 – 0.791) |
| AUC, area under the curve 95CI%,95% confidence intervals | | |

| **Supplementary Table 3. P values for AUC comparision between and pair of models tested by the DeLong method in the Budapest validation cohort.** | | | | |
| --- | --- | --- | --- | --- |
| Model (AUC) | Nomogram (0.790) | Tumor MTV  (0.640) | N stage (0.639) | Body composition (0.679) |
| Nomogram (0.790) | 1 | - | - | - |
| Tumor MTV (0.640) | 0.0474 | 1 | - | - |
| N stage (0.639) | 7.92e-06 | 0.9842 | 1 | - |
| Body composition (0.679) | 0.0789 | 0.6670 | 0.6191 | 1 |

| **Supplementary Fig.1 Flowchart of the included NSCLC patients.** |
| --- |
| 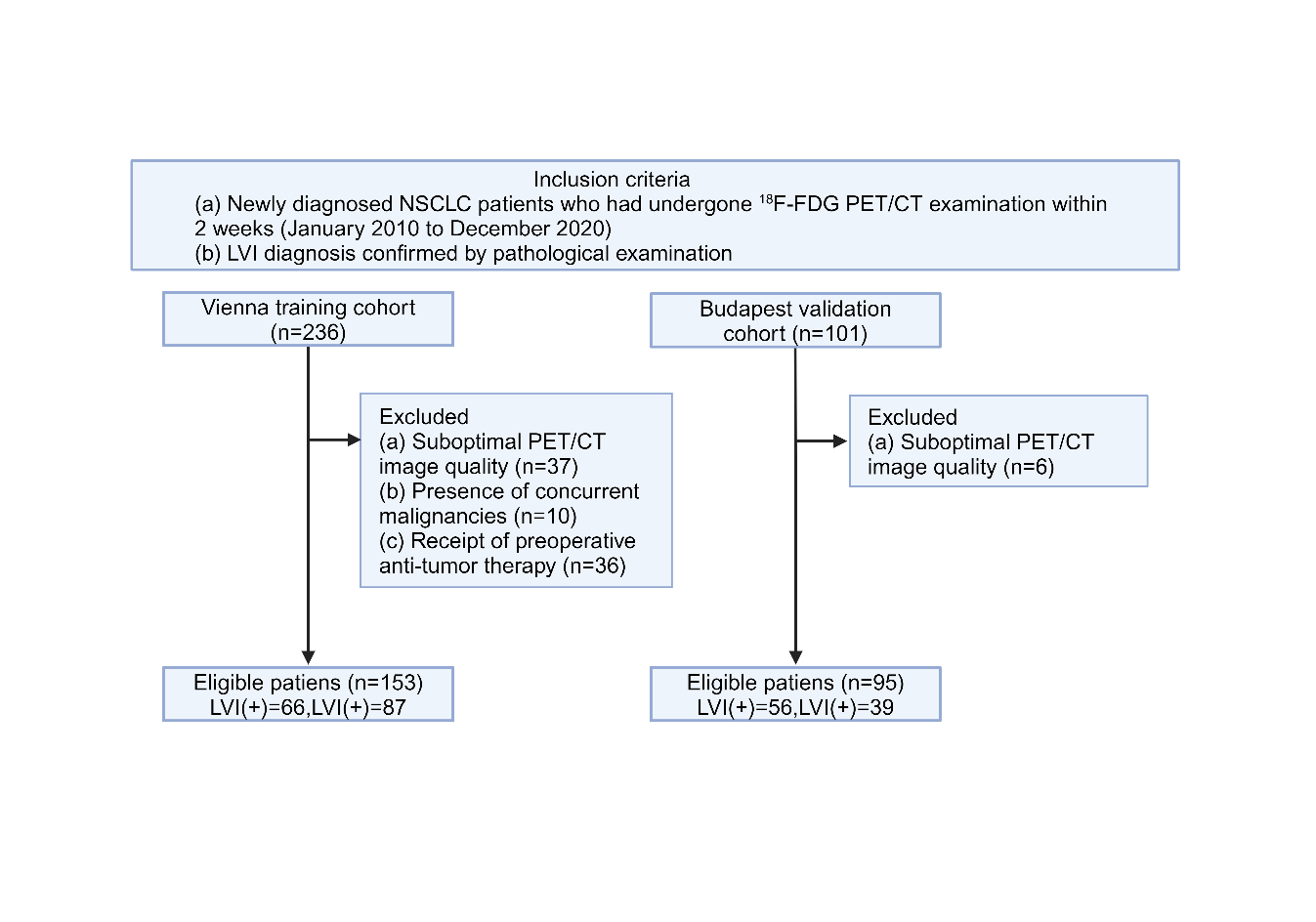 |

| **Supplementary Fig.2 Gender-specific Distribution of Body in NSCLC Patients from the Vienna and Budapest Cohorts.** |
| --- |
| 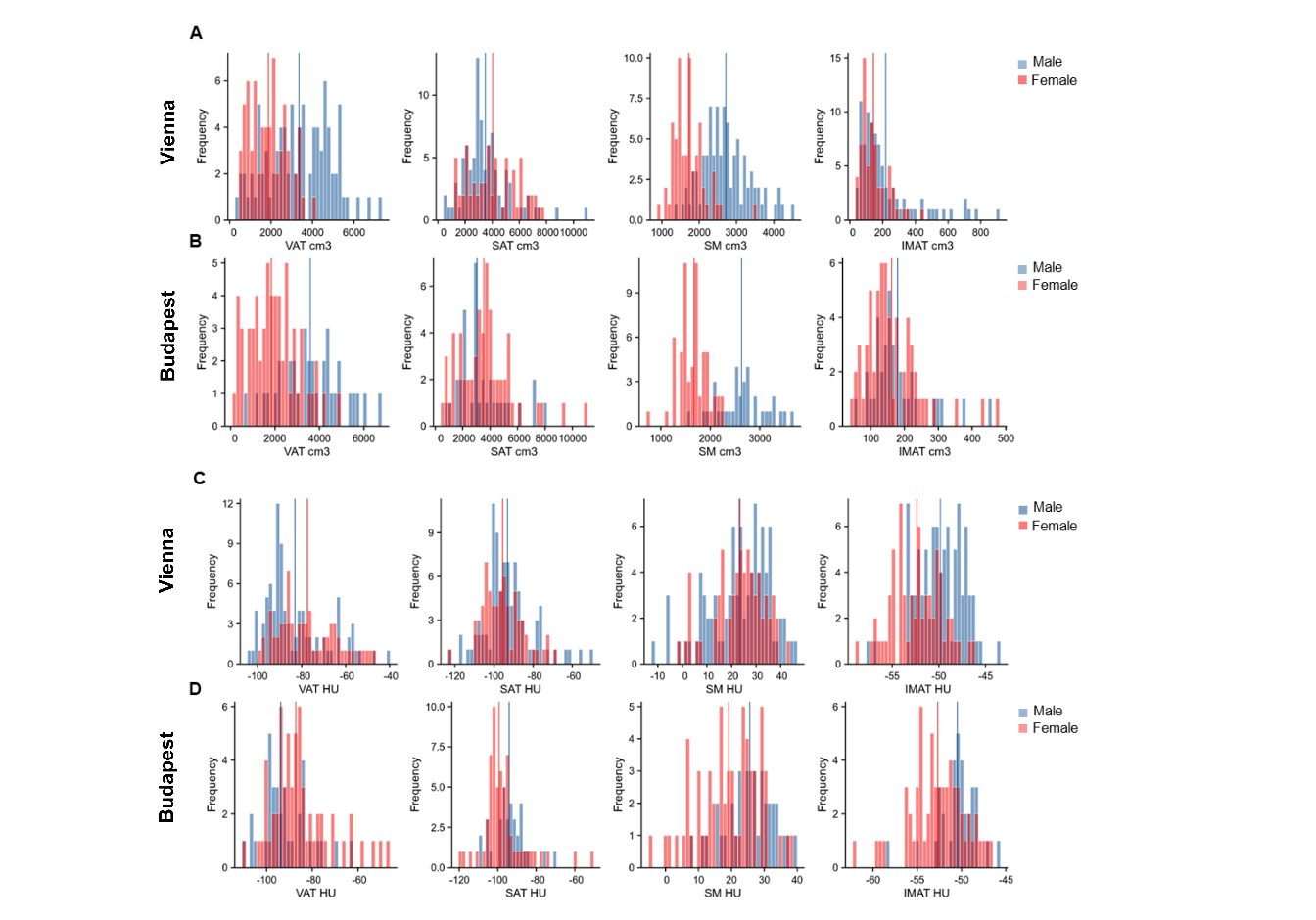  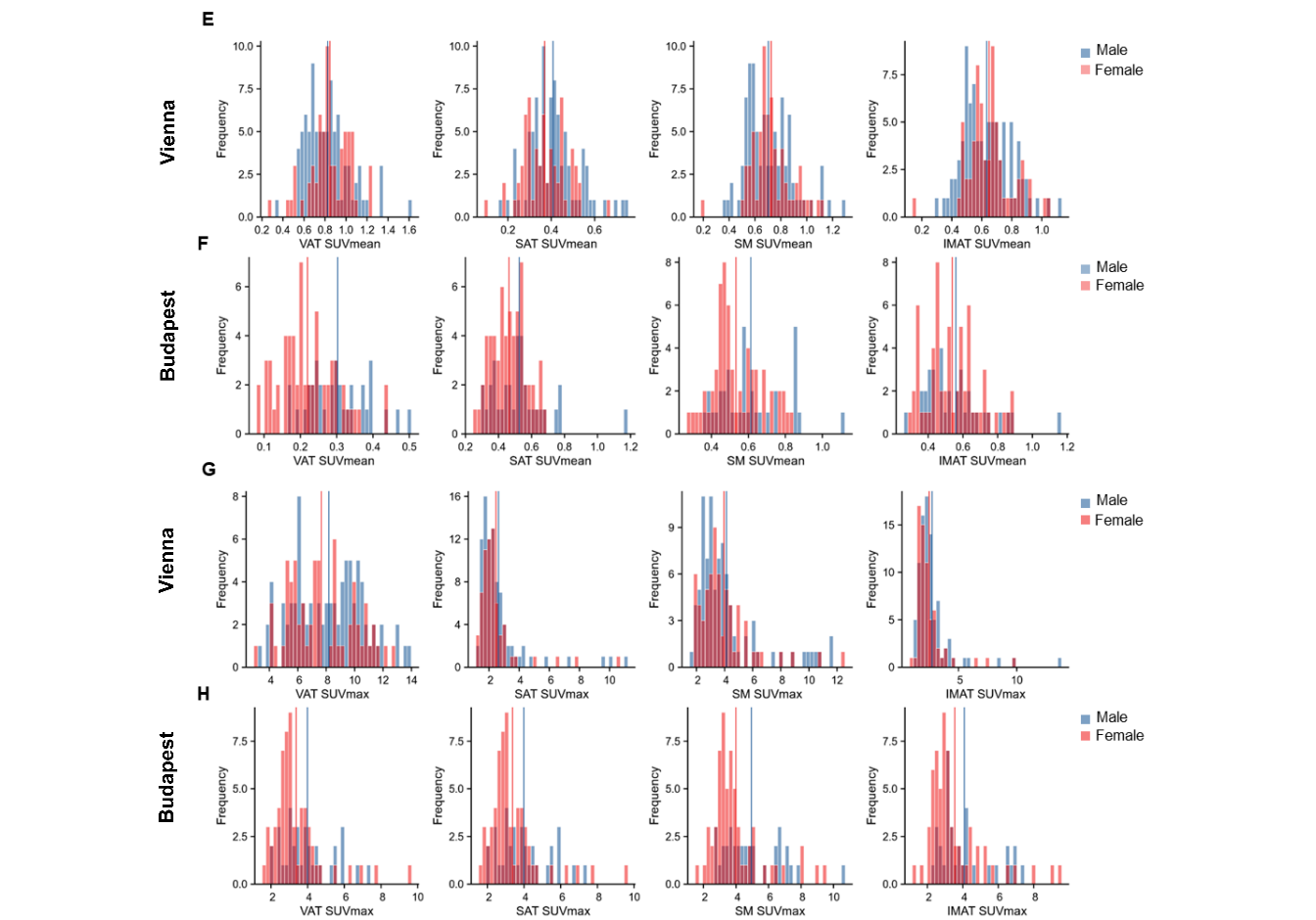 |
| Distribution of body composition at primary diagnosis NSCLC is shown by sex for the Vienna cohort (N=153) and the Budapest cohort (N=95). Vertical lines indicate the mean values for female patients (red) and male patients (blue) in each cohort. |

| **Supplementary Fig.3 Selection of influential features using the LASSO regression model.** |
| --- |
| 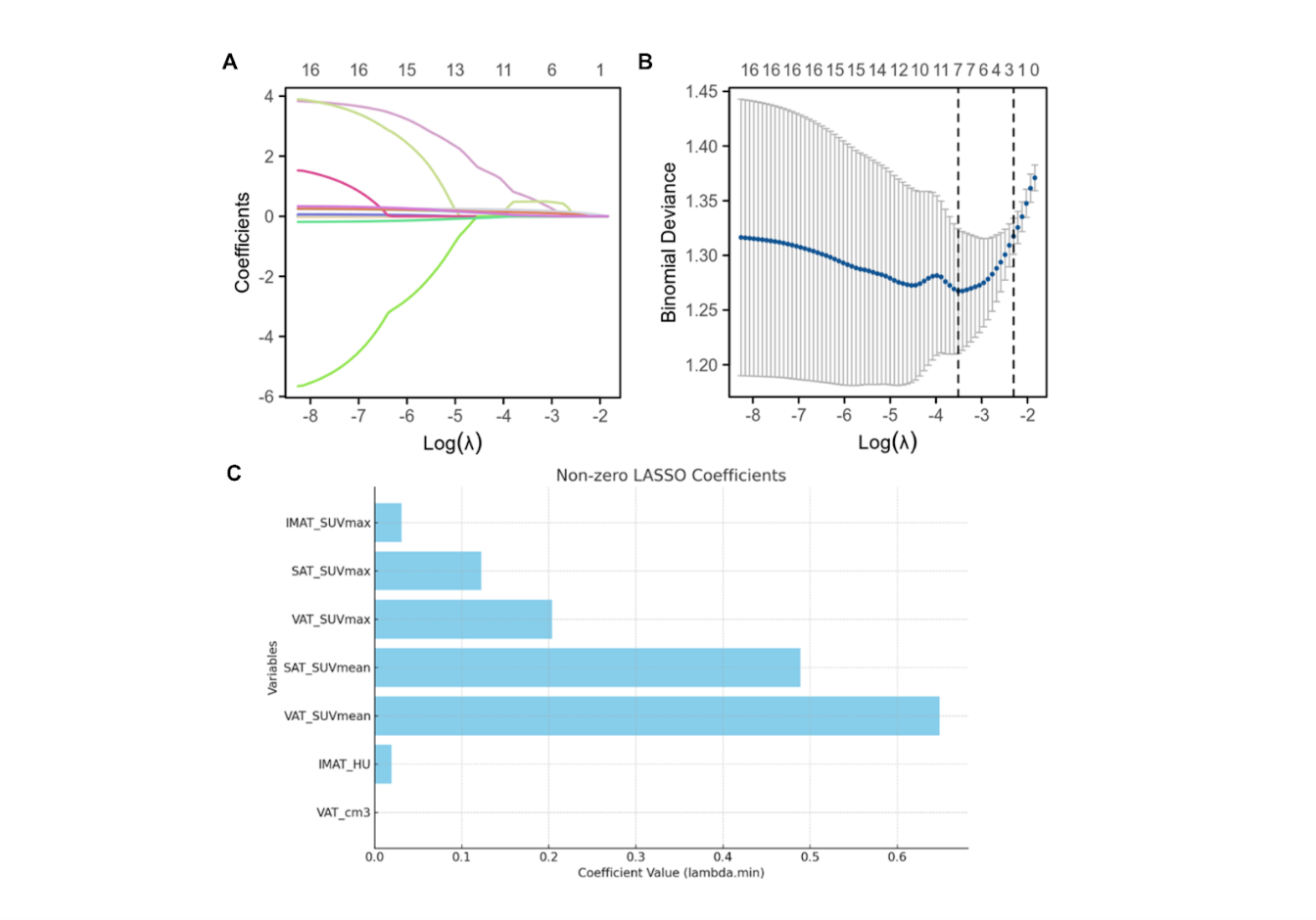 |
| (A) LASSO coefficient curves were plotted for 16 predictors. (B) The optimal penalty coefficient, lambda, was identified using 10-fold cross-validation and the minimum criterion in the LASSO regression model. The left vertical dashed line represents the minimum error, and the right vertical dashed line represents the cross-validated error within 1 standard error of the minimum. (C) The bar chart illustrates the seven selected variables with nonzero coefficients. LASSO, least absolute shrinkage and selection operator |

| **Supplementary Fig.4** |
| --- |
| 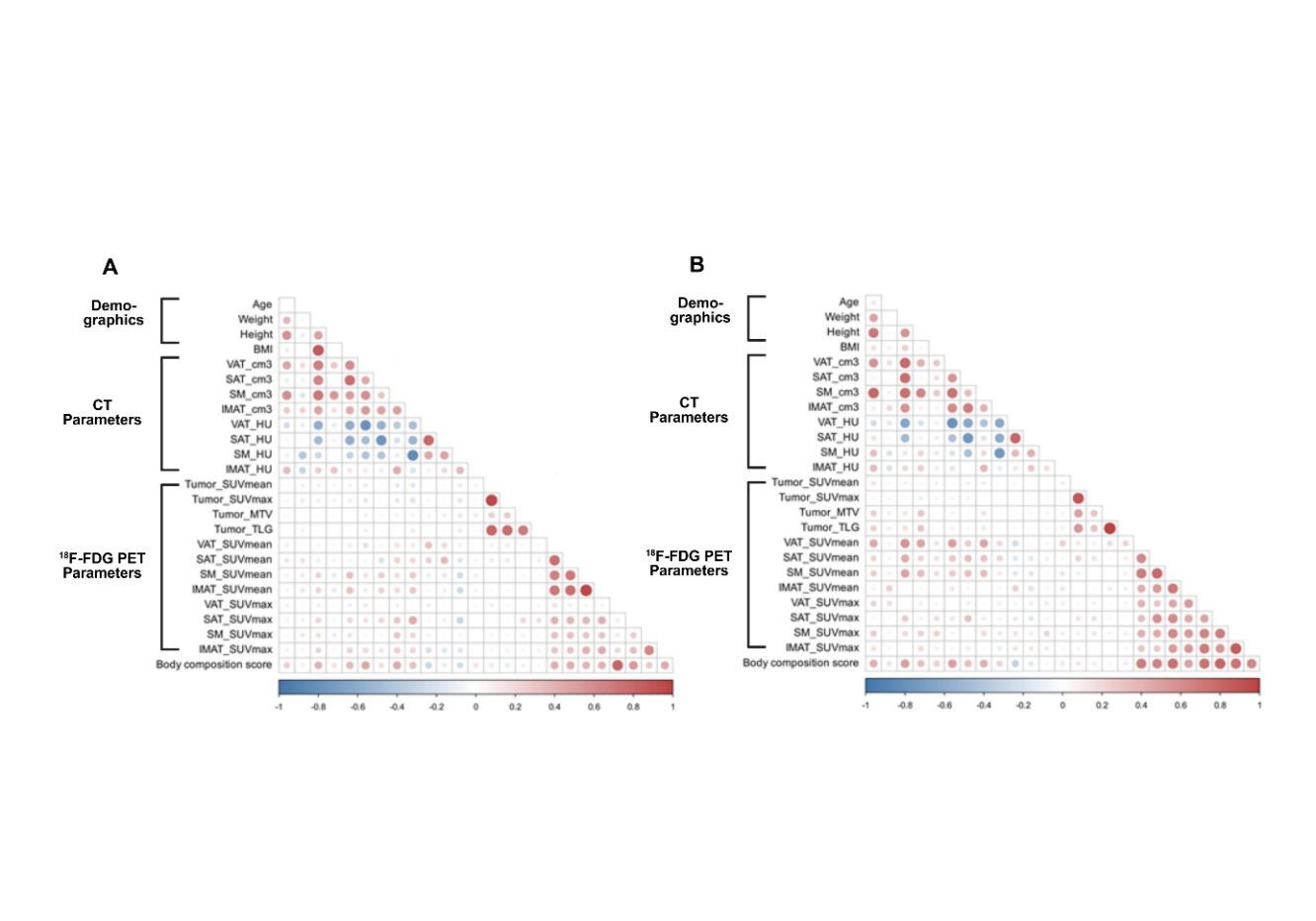 |
| Correlation matrices for the Vienna cohort (A) and Budapest cohort (B) were generated to assess the relationships between demographic factors and ^18^F-FDG PET/CT parameters. Pearson correlation coefficients are visualized, with the color and size of the circles indicating the strength and direction of the correlations—red for positive correlations and blue for negative correlations. Larger circles represent stronger correlations. |

| **Supplementary Fig.5 Body composition comparisons by histological subtypes and pleural invasion groups.** |
| --- |
| 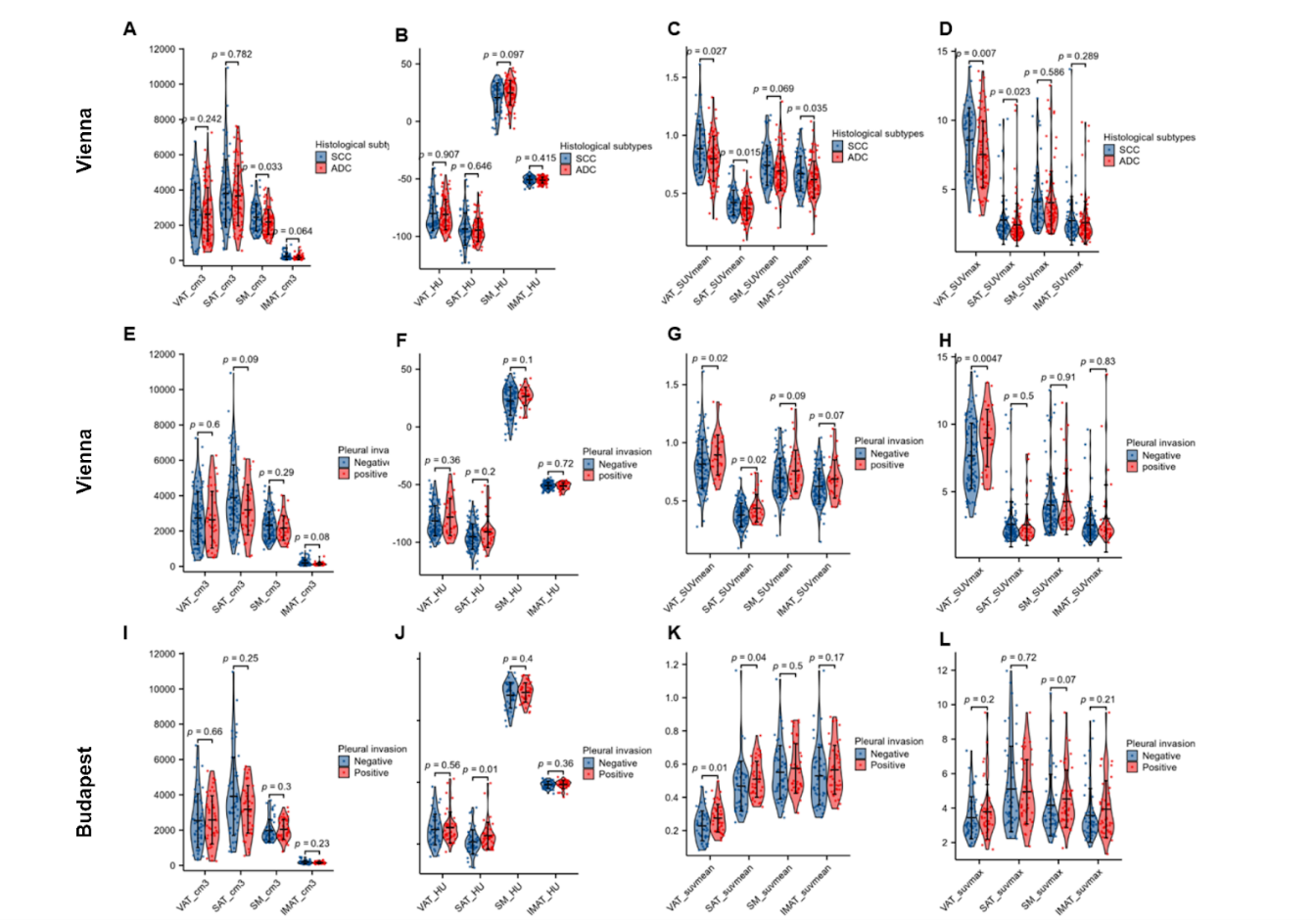 |
| Body composition comparisons in histological subtypes and pleural invasion groups. Violin plots compare body composition between histological subtypes, adenocarcinoma (ADC, red) and squamous cell carcinoma (SCC, blue), in the Vienna cohort (A–D) and between pleural invasion positive (red) and negative (blue) groups in the Vienna (E–H) and Budapest (I–L) cohorts. The measured features include visceral adipose tissue (VAT), subcutaneous adipose tissue (SAT), skeletal muscle (SM), and intermuscular adipose tissue (IMAT) derived from ^18^F-FDG PET/CT parameters: volume (cm³), Hounsfield Units (HU), SUVmean, and SUVmax. |

| **Supplementary Fig.6 Prognostic value of N stage, tumor MTV, body composition.** |
| --- |
| 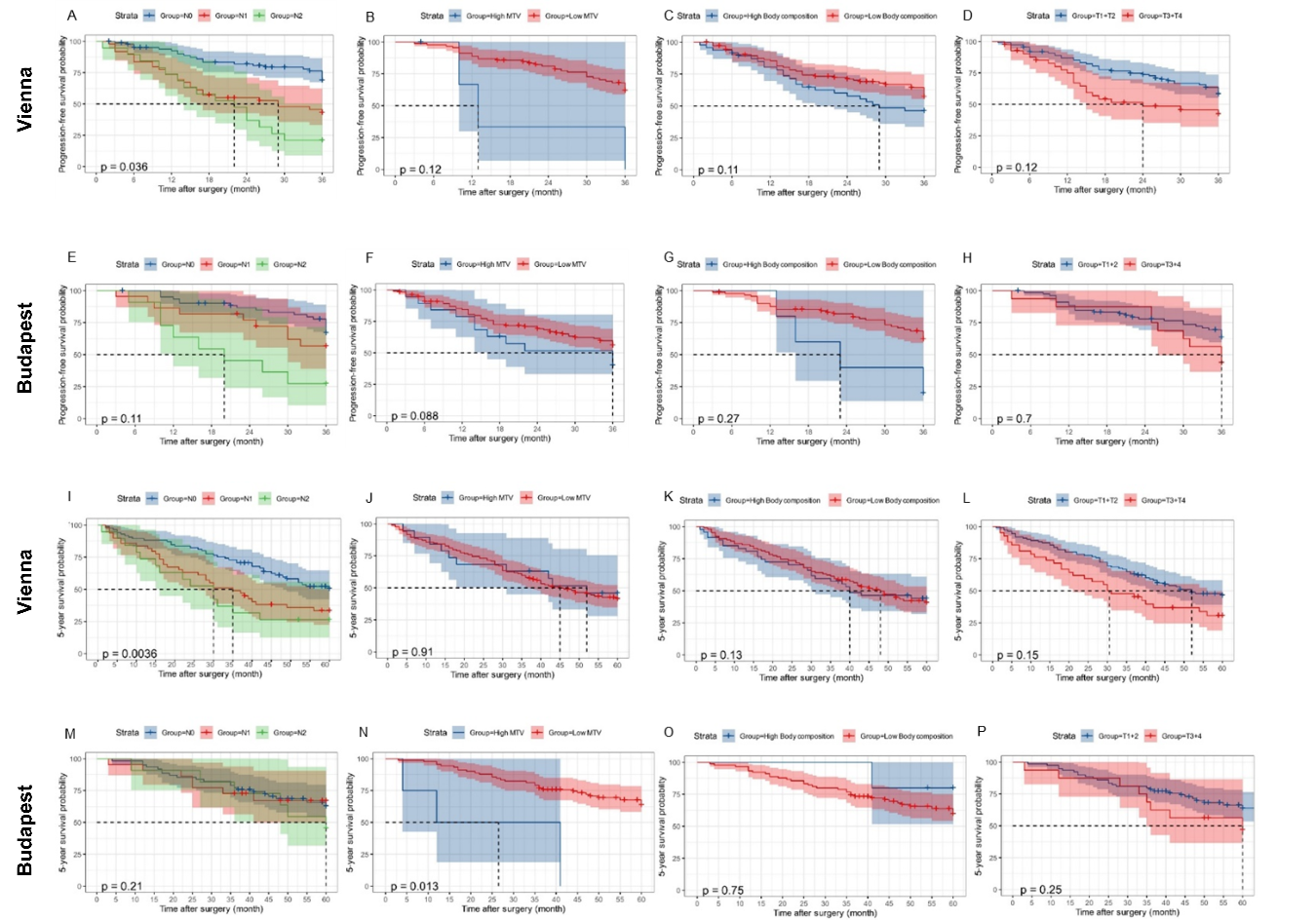 |
| The Kaplan–Meier curves illustrate 3-year recurrence-free survival (RFS) and 5-year survival in the Vienna cohort (A–D; I-L) and the Budapest cohort (E–H; M-P), stratified by pathological LVI status and LVI status predicted by the nomogram. |

| **Supplementary Fig.7 Prognostic value of T stage and LVI risk groups.** |
| --- |
| 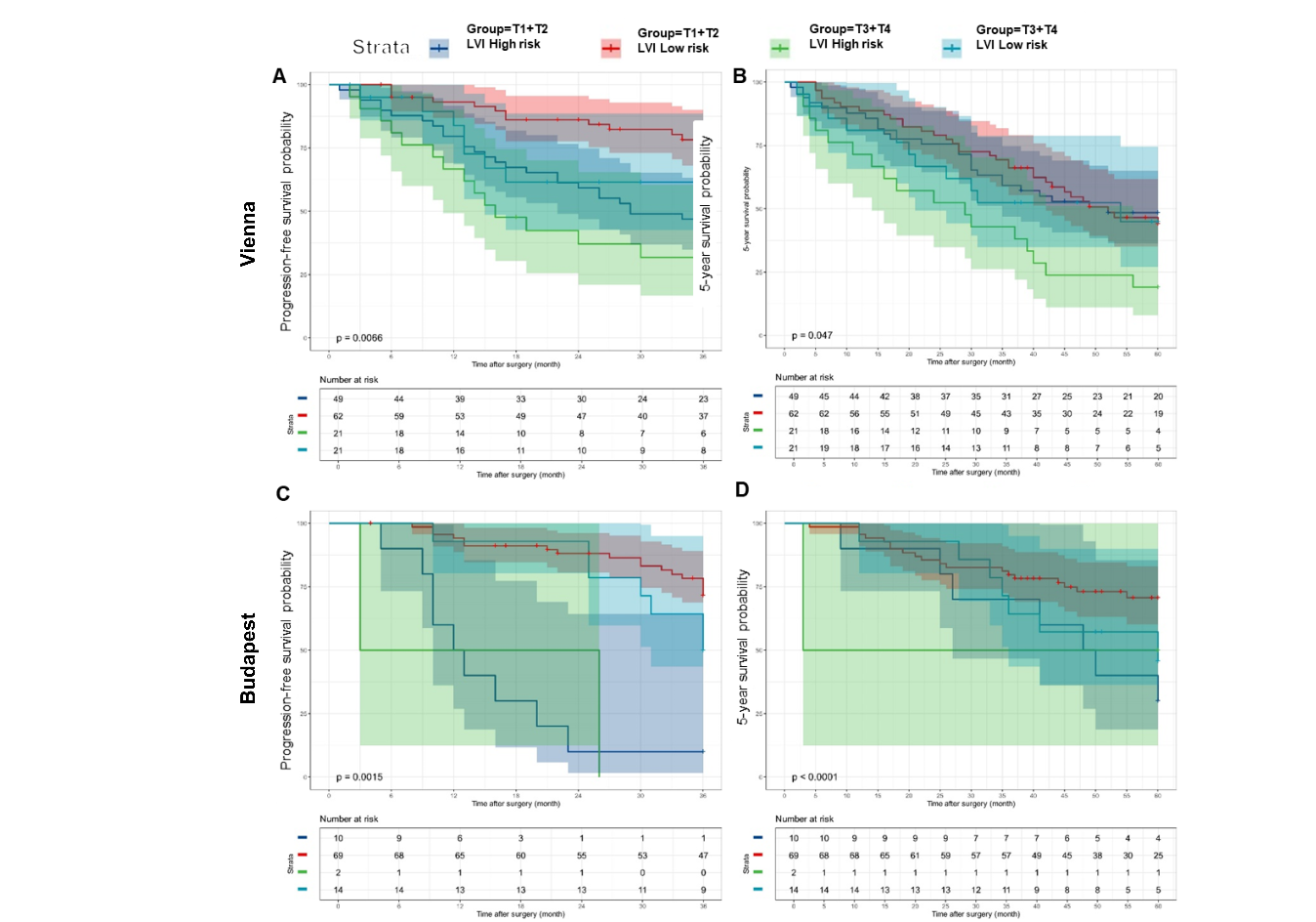 |
| The Kaplan–Meier curves illustrate progression-free survival (PFS) and 5-year survival in the Vienna cohort (A, B) and the Budapest cohort (C, D), stratified by T stage (T1+T2 vs. T3+T4) and LVI risk groups predicted by the nomogram. |

**Reference**

1.Boellaard R, Delgado-Bolton R, Oyen WJG, et al. FDG PET/CT: EANM procedure guidelines for tumour imaging: version 2.0. Eur J Nucl Med Mol Imaging 2015; 42: 328–54.

2.Fedorov A, Beichel R, Kalpathy-Cramer J, et al. 3D Slicer as an image computing platform for the Quantitative Imaging Network. Magn Reson Imaging 2012; 30: 1323–41.

3. Isensee F, Jaeger PF, Kohl SAA, Petersen J, Maier-Hein KH. nnU-Net: a self-configuring method for deep learning-based biomedical image segmentation. Nat Methods 2021; 18: 203–11.

4.Wasserthal J, Breit H-C, Meyer MT, et al. TotalSegmentator: Robust segmentation of 104 anatomic structures in CT images. Radiol Artif Intell 2023; 5: e230024.
